# Using a Particle Counter to Inform the Creation of Similar Exposure Groups and Sampling Protocols in a Structural Steel Fabrication Facility

**DOI:** 10.3390/toxics5040034

**Published:** 2017-11-23

**Authors:** James Mino, Bernadette Quémerais

**Affiliations:** Division of Preventive Medicine, University of Alberta, Edmonton, AB T6G 2S2, Canada; jmino@ualberta.ca

**Keywords:** similar exposure groups, welding fumes, occupational exposure, workplace surveys, condensation particle counter, direct reading instrument

## Abstract

The objective of this project was to create similar exposure groups (SEGs) for occupational monitoring in a structural steel fabrication facility. Qualitative SEG formation involved worksite observation, interviews, and audits of materials and procedures. These were supplemented with preliminary task-based shop survey data collected using a condensation particle counter. A total of six SEGs were formed, with recommendations for occupational exposure sampling for five groups, as well as ambient sampling recommendations to address areas on the operational floor found to have higher particle concentrations. The combination of direct reading device data and qualitative SEG formation techniques is a valuable approach, as it contains both the monetary and temporal costs of worksite exposure monitoring. This approach also provides an empowering in-house analysis of potentially problematic areas, and results in the streamlining of occupational exposure assessment.

## 1. Introduction

Occupational exposures are the fourth greatest driver of lung disease worldwide, outweighed by—but inseparable from—tobacco, household, and outdoor air pollution [1]. Welding is a suspected cause of occupational asthma, and total fume exposure has a dose responsive relationship with airway reactivity [2]. Exposure to welding fumes increases the risk of Chronic Obstructive Pulmonary Disease (COPD) regardless of smoking status, and exacerbates the frequency, duration, and severity of upper and lower respiratory tract infections [2,3]. Exposure to welding fumes also appears to lower heart rate variability and increase DNA methylation [4]. One of the primary exposures of welding is metal oxide; when welding on mild steel, iron oxides and manganese oxide are implicated in the generation of pro-inflammatory cytokines and macrophage infiltration [5]. We worked with a structural steel fabrication shop to develop a monitoring program to determine workers’ exposure to respirable metal and particulate matter. The overall objectives were: (1) to gather detailed information about job descriptions and time spent on tasks, (2) to collect task-based and area particle counts to characterize processes and backgrounds, (3) to use job descriptions and task-based/area sampling of group employees into similar exposure groups (SEGs), and (4) to make recommendations on exposure monitoring and control. In this paper, we used a direct reading instrument (particle counter) to supplement qualitative SEG creation. Condensation particle counters have been used in previous studies to characterize welding processes, but none were used to help determine similar exposure groups [6,7,8,9,10,11,12].

## 2. Materials and Methods 

### 2.1. Description of Worksite

**Figure 1:**
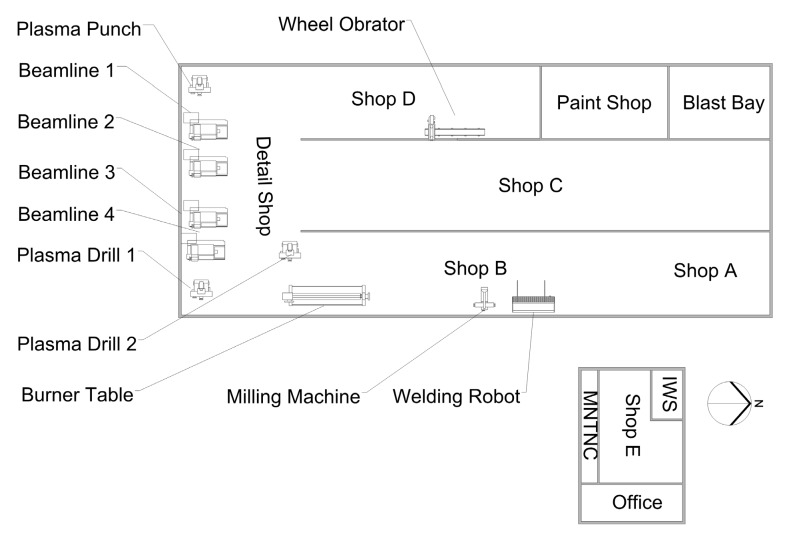
Floorplan of workshops detailing plant operation.

The structural steel fabrication shop employs around 250 full-time workers, including approximately 30 welders and welding apprentices, as well as a similar number of fitters and fitting apprentices. The site contains the following equipment for cutting, machining, and welding: computerized numerical control/personal control (CNC/PC) machinery, including one oxyacetylene/plasma (burner) table; one robotic welding machine; three plasma cutting tables; and four beamlines. Other industrial equipment of interest includes a wheel obrator and milling machine. Production occurs in two buildings, with the shop E building housing shop E, as well as maintenance and ironworker shops. The majority of production occurs in the main building, which houses shop B/A, C, D, detail, paint, and a segregated sandblasting bay. There is limited segregation between the operations: shops B/A, C, and D have partial walls with shipping doors, while at the south end of the main building, the detail shop runs east–west without segregation, with the other shops oriented perpendicularly to it (Figure 1). The facility relies on general (dilution) ventilation, and the personal protective equipment (PPE) policy mandates that welders wear respirators. When the weather is mild, doors are opened to facilitate air renewal inside the facility. All of the field work for this study was performed between January and April 2015.

### 2.2. Purchasing Records, Literature Review 

It is an accepted practice to analyze purchasing records to obtain current information on in-use consumables [13]. Purchasing records were examined to see the most commonly purchased consumables applicable to the processes and tasks under study, such as: electrodes for flux cored arc welding (FCAW), metal cored arc welding (MCAW), and shielded metal arc welding (SMAW); grinding discs, sand blasting abrasive, and wheel obrator shot. Purchasing records were received on 10 January 2015 from the Quality Control coordinator. Information obtained from the safety data sheets (SDSs) of these products were used to inform the likely exposures occurring at the construction facility [14,15,16,17,18].

### 2.3. Creation of Preliminary SEGs

The physical demands analysis (PDAs) amongst other things, provided a brief job description, a breakdown of the tasks, and approximate devotion of time to each task in a given shift/week/month, as applicable. The job descriptions (JDs) provided more details on the specific responsibilities of each position, and how the positions fit into the operational hierarchy. Personal interviews were conducted from January to March 2015 to elucidate detailed information on their job, such as: tasks with the potential to generate particulate, what shop interviewees worked in, the location of their workstation, and their work routine in relation to the shop floor. The focus was on vaguely defined jobs and junior positions, such as general helper, trade helper, and first year apprentices.

### 2.4. Quantification of Particle Number Concentrations

To determine the importance of each task on personal exposure to ultrafine particulate (UFP), a P-Trak (TSI, Shoreview, MN, USA) was used to measure particle levels. The P-Trak is a condensation particle counter with a detection range of between 20 nm and 1000 nm in size that records values in terms of particle concentration per millilitre (part/mL). The particle count threshold is 500,000 part/mL. The instrument is equipped with a probe connected to it using a 1 m-long tygon tubing. The use of the probe allowed the collection of samples at the breathing zone of workers. The instrument has two different sampling modes. In the “sample” mode, the instrument takes one reading every second for 5 s and gives an average of the five readings. In the “log” mode, the instrument takes one reading at the interval specified by the user. The instrument records data that can be downloaded using the instrument software.

### 2.5. Shop Area Sampling

Sampling was performed using the P-Trak (TSI, Shoreview, MN, USA) in “log” mode with the particle concentration recorded every second. Shop area sampling was performed as surveys to evaluate variations in particle levels in the shops, with each survey logging approximately 300 points. These surveys should be considered as background, or area, concentrations in each specific shop. The shop surveys were created by walking a pre-set path determined by landmarks in each shop to visualize any pattern of particle concentration in the shops. Since this was an active worksite, differences in survey length within shops were due to changes in walking speed, right of way, and hazard avoidance rather than changes in survey route. The survey was repeated to identify trends and hotspots in particle generation, and results were graphed for visual interpretation (see Section 3.3). The walkthroughs were performed on 17, 18, and 26 (twice) February; 23 March; and 2 and 16 April 2015. Survey sessions followed the same order: shop B/A, shop C, shop D, paint shop, detail shop, shop E, ironworker shop, and maintenance shop. 

### 2.6. Task-Based Exposure Data Collection

The particle number concentration was measured in the breathing zone of the workers while they performing the task [19] by setting the P-Track to “sample” mode, as previously described. Only tasks that were likely to result in significant particle release were selected. Therefore, tasks such as crane operation were not evaluated, since there was no expectation that crane operation would generate particles. Thus, the crane operator’s exposure could be attributed to background levels. Sandblasting and spray painting were not evaluated for safety reasons, and to avoid any damage on the particle counter. Task-based sampling [20] included the following manual processes: disc grinding, SMAW, FCAW, MCAW, air carbon arc cutting (gouging), and oxyacetylene cutting (oxycutting). Task-based sampling was also performed on the operators of the following equipment: the four beamlines, the one plasma drill, the one welding robot, the one milling machine, and the one burner table. Processes were sampled on 18, 19, and 27 February; 17 and 20 March; and 2 and 16 April 2015. Not all processes were sampled on all days. 

All samples were taken in the breathing zone; however, when samples were taken was determined by the particulars surrounding an operator’s duties. The plasma drill samples were taken while the operator was approaching the cutting surface, rather than at his station. It was observed that for small work pieces, the operator had to approach the cutting surface to mark them with a paint pen while the plasma torch was engaged, and removed the finished pieces from the table by hand, which was unique amongst CNC/PC operators. Samples taken from the operators of the burner table and milling machine were taken exclusively at the operating station, because the operator did not approach the machine during operation. In the case of the welding robot and the beamlines, samples were taken with the operator as they occupied the station and as they entered the work area, because they often entered the work area to ensure the program was running correctly (welding robot), or to label the pieces (beamline), but in neither case were they able to manually handle the material, unlike the plasma drill operator. 

### 2.7. Statistical Analysis

Statistics were performed using GraphPad Prism (GraphPad Software, Inc., La Jolla, CA 92037, USA). Since data were not normally distributed, non-parametric tests such as the Krustal–Wallis test and Dunn’s multiple comparison tests were used on non-transformed data. Significance was accepted at *p* < 0.05. Graphs are presented with the geometric mean and 95% confidence interval of the geometric mean.

## 3. Results

### 3.1. Preliminary SEGs

Preliminary exposure groups are shown in Table 1.

### 3.2. Shop Area Sampling

**Figure 2:**
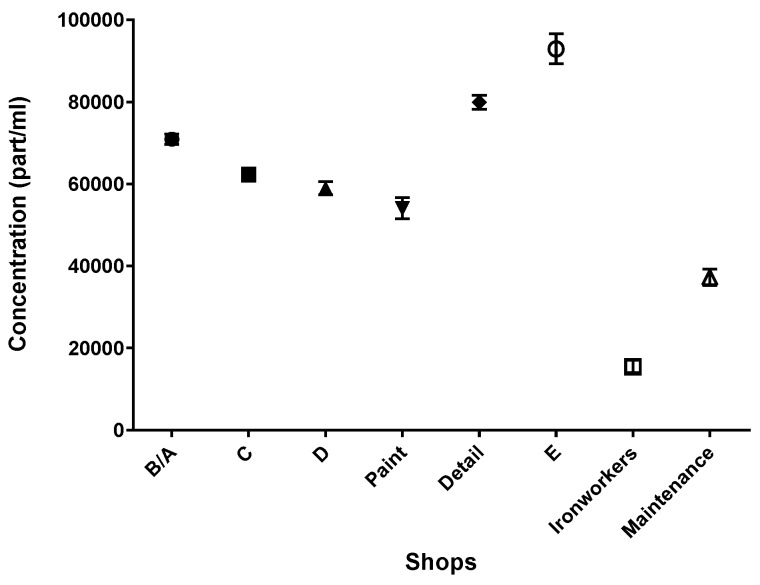
Geometric means (GM) and 95% confidence intervals of shop surveys; B/A (*n* = 2200), C (*n* = 2085), D (*n* = 1435), paint (*n* = 757), detail (*n* = 2732), E (*n* = 1205), ironworkers (*n* = 538), and maintenance (*n* = 630).

The particle counts logged during the shop surveys were combined per shop, and are displayed as a graph in Figure 2. Note that the number of samples per shop varied from one shop to another due to the various length of the pathways. Inexplicably, there were no indications that the P-Trak could detect the paint aerosols, since particle counts from surveys conducted while spray painting was being performed were indistinguishable from surveys conducted when spray painting wasn’t being performed.

A Kruskal–Wallis multiple comparison showed that the particle counts between all shops were significantly different (*p* < 0.001), except for shop D and the paint shop (*p* = 0.0886). The detail shop and shop E showed the highest background particle counts, with geometric means (GMs) of 79,900 and 92,900 part/mL, respectively. The ironworker and maintenance shops showed the lowest background particle count, with GMs of 15,400 and 37,200 part/mL, respectively.

#### Shop Area Sampling Profiles

**Figure 3:**
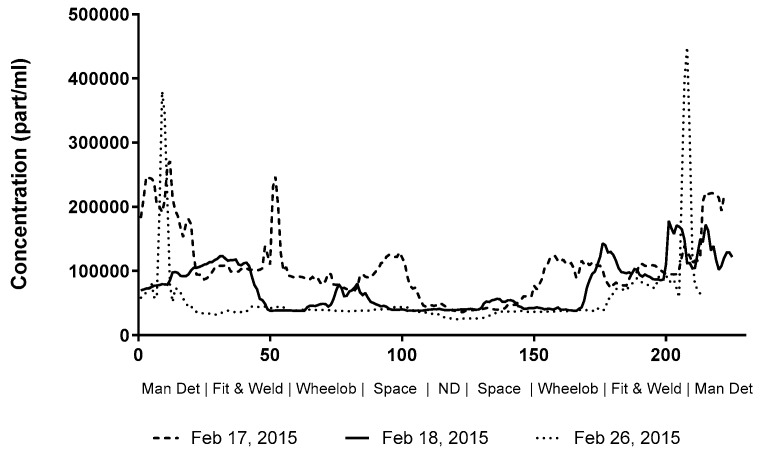
Selected shop D data.

Individual shop profiles were created for the shops housed within the main building and the shop E building by aligning surveys according to their landmarks and superimposing each walk-through for comparison. Only the results from shop D are shown. The path in shop D began at the south end of the shop and traveling northerly until reaching the paint shop doors (ND) and then returning to the starting point; landmarks are listed sequentially as encountered on the survey path. There was an obvious elevation of particles bookending the survey, which aligned with the manual detail section. By selecting out the three surveys with comparable run times, a horseshoe pattern emerged, as illustrated in Figure 3, which reflected the disproportionate amount of particulate that the manual detail area generated, with the lowest particle counts at approximately 30,000 part/mL, and the highest over 400,000 part/mL.

### 3.3. Manual Tasks

**Figure 4:**
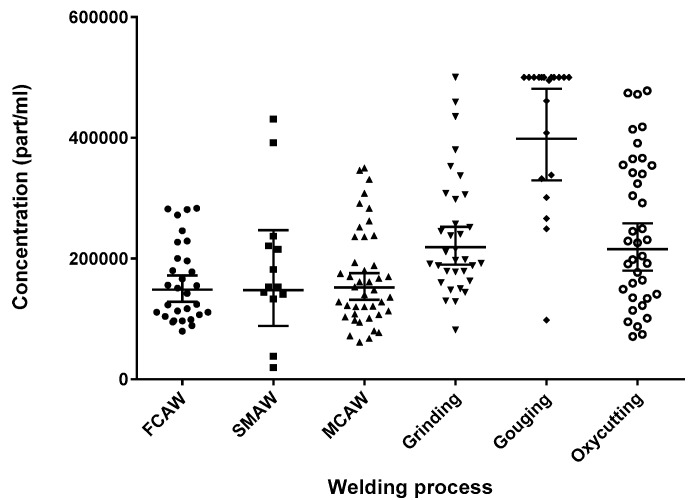
Manual task breathing zone grab samples; FCAW (*n* = 30), SMAW (*n* = 13), MCAW (*n* = 42), grinding (*n* = 33), gouging (*n* = 20), and oxycutting (*n* = 38). FCAW: flux cored arc welding; SMAW: shielded metal arc welding; MCAW: metal cored arc welding.

Results for manual task grab samples are shown in Figure 4. Recall that each sample point is the average of the per-second readings taken for the 5 s sample length. Manual task sampling had two notable limitations: taking valid SMAW samples, and instrument limitations biasing gouging results. SMAW is a welding process used exclusively by fitters to tack pieces in place. It was difficult to sample, because tacking often lasted just a few seconds. This meant that arc ignition had to be anticipated in order to get a valid sample, which made these samples less reliable than other samples taken. Gouging generates far more particulates than other processes, and was limited by the upper detection limit (ceiling) that the P-Trak can register of 500,000 part/mL, resulting in an underestimation of the particle concentration produced by this process. With these limitations in mind, a Kruskal–Wallis test determined that FCAW, SMAW, MCAW, grinding, gouging, and oxycutting generated significantly different particle levels (*p* < 0.0001).

Dunn’s multiple comparison test showed that all three welding processes were not significantly different (*p* > 0.99). Interestingly, grinding and oxycutting, the two sources of exposure that are most common amongst fitters as determined by work observation, were highly similar (*p* > 0.99). Gouging generated the most particulate, which was significantly higher across all comparisons (*p* = 0.0053 to *p* < 0.0001). Welders routinely grind, and it was found that grinding generated more particulate than FCAW (*p* = 0.04).

### 3.4. Machine Exposures

**Figure 5:**
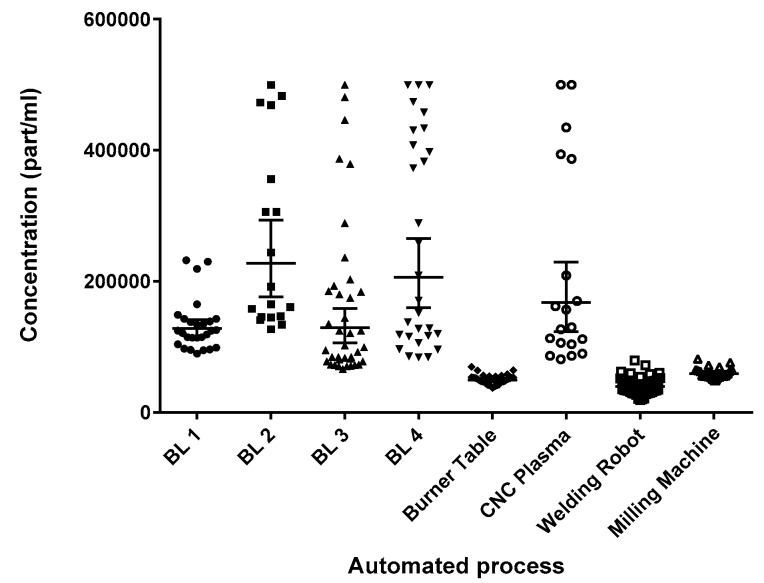
Distribution of machine source breathing zone grab samples; BL 1 (*n* = 27), BL 2 (*n* = 18), BL 3 (*n* = 38), BL 4 (*n* = 29), burner table (*n* = 35), CNC plasma (*n* = 19), welding robot (*n* = 47), and milling machine (*n* = 23). BL: beamline; CNC: computerized numerical control.

Results for all of the machine operator samples are shown in Figure 5. As for manual tasks, each point represent the average of five readings taken over 5 s. A Kruskal–Wallis test demonstrated that the sources generated significantly different particle levels (*p* < 0.0001). 

Using Dunn’s multiple comparison test, the beamlines were not different from CNC plasma cutting (*p* > 0.99). The welding robot, milling machine, and burner table had very low particle generation; these levels were comparable to the median shop B/A background levels, and were significantly different than all other processes (*p* < 0.005), but not significantly different from one another (*p* > 0.1). 

**Figure 6:**
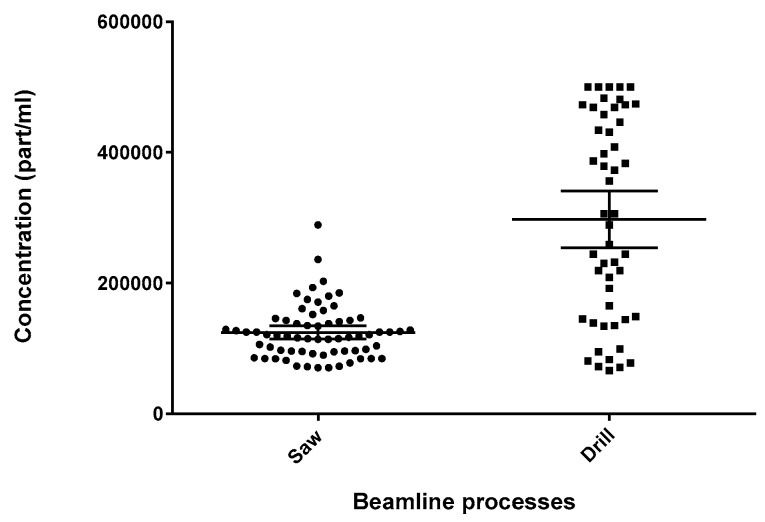
Beamline processes grab sample distribution; saw (*n* = 66) and drill (*n* = 50).

A Kruskal–Wallis test on the four beamlines showed that they too generated significantly different particulate levels (*p* < 0.0001) with beamlines 1 and 3 (BL 1 and BL 3) being significantly different than beamlines 2 and 4 (BL 2 and BL 4). However, there are two processes on each beamline—sawing and drilling—and using the Mann–Whitney U-test on the pooled values from all beamlines, it seems that drilling produced more particulate than sawing (*p* < 0.0001) (Figure 6).

Therefore, the finding that the beamlines generated significantly different particle counts may be an artifact of the difference in the number of samples collected for each process (saw versus drill) from each of the four beamlines.

### 3.5. Workers’ Exposure

To evaluate workers’ exposure, results from manual and automated processes as well as results from shop surveys were combined with the job descriptions and field observations. Detailed information about each job is described in Table A1. Table 2 presents the geometric mean and 95% confidence intervals for various activities, as well as the shops used to estimate workers’ exposure. Although SMAW process had four outliers—two very low points and two very high points—all welding processes showed similar geometric means (Figure 4) and were combined, since they were not significantly different. There was a large variability in particle generation between the beamlines. However, as discussed, this variation is likely an artifact of multiple processes, and further, no significant difference was found between the beamlines and the CNC/plasma. With concentrations ranging from 37,600 to 500,000 particles/ml (Figure 5), it was decided to combine all of the data for beamlines and the CNC plasma to have a CNC/PC operator. However, the geometric mean might not be really representative of the true exposure. Only full shift monitoring will allow a better estimate of exposure. Since the shops are all connected, and since geometric means were similar in shops B/A, C, and D, all of the data were combined.

Particle concentration per task was calculated by multiplying the percentage of time spent in a task to the geometric mean of that particular task. When the percentage of time on task was below 100%, the remaining time was multiplied by the geometric mean of the shop where the workers were located, and considered as background concentration. For drilling, the geometric mean for the drilling operation from the beamlines was used, since we did not have it as a manual task. For job titles with no particle-generating tasks, the geometric mean of the shop where the workers were located was used (i.e., background concentration).

Note that activities such as sandblasting and painting were not properly evaluated, and are considered as not measured. Estimated concentrations were calculated by adding all of the tasks per job title.

The percent of time attributed to each task per job, associated particle concentration per task (i.e., percentage spent on task x particle count per task, from the data given in Table 2), as well as estimated concentration per job (i.e., the sum of each task) is shown in Table 3. Interestingly, helpers and fitters were as exposed to particles as welders, since they spent more time performing tasks generating high particle levels such as drilling and grinding. For the same reason, the CNC/PC operator showed high exposure levels, since the automated processes from the beamlines and CNC plasma also produced high levels of particles.

## 4. Discussion

Background concentrations obtained from the shop surveys were in line with both observations and knowledge of the fabrication shop operations with the overall background levels attributable to the reliance on dilution ventilation in a high particle-generating environment. The significant differences between shops is in agreement with the asymmetric distribution of workload, machinery, and manpower. Shop B/A produces the largest structures that require higher weld deposition levels; has the most experienced staff, and houses the welding robot, which may explain its higher geometric mean in relation to production shops C and D. The detail shop houses virtually all of the CNC/PC machinery, which has been shown to generate a substantial amount of particulate. Shop E levels reflect the denser employee spacing, lower ceilings, and older ventilation system. The manual detail section of shop D, which is referred to in Figure 6, is where many particle-generating tasks occur, including tacking, grinding, and oxyacetylene cutting. Operationally, it has many employees working in close quarters, which is afforded by the comparatively small size of the workpieces; the consequence is that exposure to particulate generated by adjacent activities is more likely than in other areas. For these reasons, the manual detail section has been selected for ambient air sampling. In general, our findings on high background levels are in agreement with previous studies done in welding training facilities [11] and various workplaces [6,12].

Manual task findings showed that grinding generated more respirable particulate than welding, and similar particulate levels to oxyacetylene cutting, which is in agreement with other findings showing thermal cutting exceedances over welding [11,12,20]. Particle concentrations obtained in our study for SMAW, FCAW, and MCAW are in the same range of what was observed in previous studies [6,8,11,12].

The depressed numbers seen with the welding robot, burner table, and milling machine are reasonable; the welding robot positions the material about five feet from the ground, and uses the MCAW process, requiring all welds to be performed in the flat (1G/1F) position. Therefore, the material being welded creates a floor, and the thermal vectors direct the particles upward, which is away from the operator in this case. The milling machine drills holes with a very slow rotational speed; the slower the drilling speed, the lower (and larger) the particle generation [21]. In addition, the use of copious amounts of cutting fluid acts to contain generated particles. The burner table is a water table-type cutting machine, which is designed to mitigate particle release.

Analogously, the CNC plasma generated similar particulate levels as the beamline. That the beamlines, which perform the same task, were significantly different is likely due to the unequal number of samples per process (i.e., sawing versus drilling) that was taken for each machine. Other possible explanations include machine model (size/specifications) differences and machine setup (position of the operating station relative to the processes) differences. We found no difference between welding processes, which deviates from the literature [22]. This may be attributable to the similarity in profiles below one micron—which is the size limit of the P-Trak—between SMAW, FCAW, and GMAW [22]. It may also be due to the conservative nature of non-parametric analyses, and/or the difficulty in sampling SMAW welding. Grinding, gouging, and oxycutting produced higher particle levels than any welding tasks, therefore putting fitters and helpers at risk for exposure to metal particles.

In welding fume, vaporized metal condenses, forming oxide particles that are primarily of respirable size, and often in the ultrafine range [19]. Welding fume is composed largely of consumable rather than base metal [23], although fume constituents are affected by welding parameters, including shielding gas, polarity, potential difference, and current [2,23]. For example, concentrations of particles during metal active gas welding (MAG–aka GMAW) were shown to increase with increasing amperage and/or increasing CO_2_ in the gas mixture [24]. Exposure is also dependent on the relative position to the weld, the volume of space, and ventilation [23]. For example, monitors distanced vertically from the weld had orders of magnitude greater deposition than monitors distanced horizontally [25], as thermal vectors channel particles upwards; also, welders in confined spaces have poorer lung function than those welding in open air [2]. The welding parameters used at the fabrication shop are quite uniform, with direct current electrode positive (DCEP) polarity and a 3:1 argon:CO_2_ gas mixture held constant. Also, consumable Safety Data Sheets (SDS) show that FCAW and MCAW electrodes are largely indistinguishable, citing manganese, iron oxide, fluorspar (CaF_2_), and amorphous silica as hazardous constituents [17,18]. In this fabrication shop, welders using the MCAW process may have a higher exposure potential due to the flat (1G/1F) position required with this consumable; beyond this, welders are likely to experience similar exposures. 

The SDS for SMAW electrodes indicate that they contain quartz silica in minute quantities [26], potentially posing a risk to fitters; however, worksite observations showed that welding is both brief and sparse. Therefore, between the low amount found in the consumable and the limited volatilization, it would seem highly unlikely that quartz silica exposure would exceed the Time-Weighted Average (TWA) of 0.025 mg/m^3^ [27]; however, without occupational sampling, this remains speculation. The SDS for the grinding discs were uninformative about whether phenolic resin particulate is of greater concern than nuisance dust [14,15]. There is some evidence that phenolic resin dusts can impair lung function [28]; therefore, it merits investigating how much abrasive dust exposure exists for welders, trade helpers, general helpers, and fitters.

When generating SEGs, it is important to remember that exposure is dependent on a number of factors, including process, material used, ventilation, and intermittency of work [20]. The welding processes, mechanical and thermal cutting methods, and abrasive polishing techniques all generate particles of concern. Therefore, the most hazardous task is not necessarily the greatest source of exposure. An example of this is with fitters, who do weld, but do so infrequently and discontinuously. Therefore, the majority of exposures likely come from mechanical and thermal cutting, which is an exposure profile that they share with trade helpers and, occasionally, general helpers. All of the thermal cutting methods generate metal oxides of the base metal [29]. Plasma cutting, and the grinding and drilling of mild steel all create a significant amount of respirable particulate, with 42% of particulate generated below 2 µm, and grinding and drilling producing a full third and 20% below 1 µm, respectively [30,31]. This presents a challenge, as plasma table operators are likely to have a similar amount of exposures as other beamline operators, and yet are exposed to the oxides of metal, rather than elemental metal, and oxidation states do affect toxicodynamics [32,33]. In practice, the Alberta Occupational Health and Safety Code does not distinguish between elemental and oxides of iron or manganese [27] which makes mechanically and thermally generated metal exposure comparable, and allows plasma drill and beamline operators to belong to the same SEG. 

In contrast to welding fumes, particulate from grinding tends to remain close to the ground, as there are no thermal vectors to displace them upwards [34]. Thus, the beamline operations may only be a regional hazard and contribute less to the ambient particulate found in the shop. It is our opinion that CNC/PC stations do not require near field air sampling, because operators’ personal samples would act as a surrogate, based on work practices. The same logic could be applied to the workers in the manual detail section of shop D, and shop E workers. However, as they perform a great number of manual tasks, it would be wise to distinguish between ambient and task exposures. Thus, both shop D and shop E should undergo ambient sampling to address the survey data observed in shop D, and the overall particulate level in shop E. Finally, to address concerns voiced by the shippers and the occupational nurse about the sandblasting abrasive spreading beyond the blast bay, near field air sampling should be undertaken in the shipping section of shop C, directly east of the wall separating the blast bay from shop C, as well as the paint shop. Finally, in our view, there is no need to perform near field air sampling in the main building shops, because repurposing personal air samples from positions classified as indirect ambient would be more representative of the overall exposure, as determined by worksite observation. 

### 4.1. Limitations

There are limitations to this study, since the P-Trak is uninformative of the true nature of the particles being counted. The instrument itself functions well in comparison to larger condensation particle counters when used indoors; while counts differ, the correlation between the P-Trak, the Scanning Mobility Particle Sizer (SMPS), and mini-disc particle counters is considered good [35]. The P-Trak can underestimate freshly emitted particles from combustion sources, and underestimates counts at the low end (<40 nm) of the detection range [35]; primary welding particles are generally lower than 50 nm in diameter, although agglomerates form with all welding processes [36]. Therefore, the measurements taken, including welding, oxyacetylene, and plasma cutting, may be underestimates of actual particle exposure. With regard to shop comparisons, although thousands of data points were logged, they result in minutes of total sampling time per day. Consequently, it would be premature to consider the ranking of shop particle concentrations found here as conclusive, and gravimetric comparison is recommended. However, the use of the instrument was useful in determining final exposure groups, and presented a fast and cheap way to estimate exposure levels. The likely chemical composition of particles can easily be determined from work descriptions and field observations.

### 4.2. Similar Exposure Groups and Monitoring Program

Similar exposure groups are simply groups of workers sharing similar exposures and frequencies; the classification serves to create a larger pool from which to sample. Preferred candidates are the positions with the most ideal fit into their respective class, which require the least amount of verification to ensure they are appropriately classified on a given sampling session, and have been listed here for illustrative purposes. The complete employment roster is assigned in the program supplied to the fabrication shop (not shown). Final SEGs are presented in Table 4, and include a range of particle concentrations, as well as the type of exposure for each group.

Exposure classification was determined as (1) Very Low to Low Exposure, with negligible exposure to mixed particles (group 1); (2) Medium Exposure, with exposure between 10,000 and 70,000 part/mL to either mixed (group 2) or metal particles (group 3); (3) High Exposure, with exposure above 70,000 part/mL to metal particles (group 4); and (4) Specific Exposure, which includes spray painters (exposure to solvents, group 5) and sandblasters/descaling operators (exposure to crystalline silica, group 6). Preferred candidates were determined as follows:

- group1: Production Manager, Corporate Maintenance Manager, General Foreman, Building Maintenance Operator, Mechanics, Shipping Clerk, Yard Man, Truck Driver, Mod Yard Crane Operator
- group 2: Lead Hand/Foreman and Ironworker
- group 3: Electrician and Shipper
- group 4: Welder, Fitter, Trade Helper, CNC/PC Operator
- group 5: Spray Painter
- group 6: Sandblaster

It is recommended to collect personal exposure samples from preferred candidates from groups 2 to 6. In addition, area samples should be collected from the shop C shipping area and paint shop to monitor crystalline silica from sandblasting operations, as well as the manual detail section of shop D and shop E, due to their high particulate levels.

It is preferable to perform sampling six to 10 times yearly for a minimum of eight hours, since shifts are 10 hours long. Due to their high exposure or exposure to very toxic chemicals, groups 4 to 6 should be sampled 10 times a year to ensure monitoring of the worst case scenario. Groups 2 and 3 can be monitored less frequently (i.e., six times a year). Note that the exposure groups can be refined once regular sampling is performed, since this work gave us only broad estimations of exposure. Refining SEGs from both a between-group and within-group perspective allows for more tailored exposure interventions and, eventually, reduced sampling requirements. Samples collected in the shipping area of shop C and from the sandblaster/descaling operator should be analyzed for crystalline silica. Samples collected in the paint shop and from the painter should be analyzed for crystalline silica, metals, and solvents to account for exposure from blasting bay, paint, and background metal particles. All other samples should be analyzed for metals and particulate.

The facility does not have any local exhaust ventilation, and uses only general dilution ventilation. Currently, the company relies upon PPE to reduce exposure to hazardous particles. The sandblaster is required to wear a helmet-style supplied air respirator (SAR); the spray painters are required to wear half-face air-purifying respirators (APRs) with gas/vapour cartridge filters; and the welders are required to wear a half-face air-purifying respirators (APRs) with P100 (oil proof HEPA) filters. However, the PPE policy does not mandate that other personnel wear any respiratory protection. Our recommendations are to have all members of SEG group 4 wear a half-face air-purifying respirators (APRs) with P100 filters. It was observed that the sandblaster would remove his helmet immediately after abrasive blasting; it ought to be policy that he or she would not be allowed to remove the helmet while in the blast bay. Although installing a local exhaust ventilation system (LEV) would be very costly, it might be interesting for the company to investigate using portable extractors for welding fumes, which should help reduce the background levels in the shops.

## 5. Conclusions

Using a direct reading instrument such as a P-Trak particle counter is an excellent way to add preliminary quantitative evidence to the development of a sampling strategy. Additionally, the same instrument is an effective way to map or survey a space for developing spatial inferences for the sampling strategy in question. In addition, our results showed that trades such as fitters and helpers can be exposed to levels of particles as high as welders, and should not be omitted in a monitoring program, especially since they are often not mandated to wear respiratory protection. Similarly, machine operators may be at risk of high exposure if the machine they operate uses a high particle-generating process, such as drilling or thermal cutting. It is interesting to note that many studies concern welders, while other trades might be as much at risk as welders for respiratory diseases. In the case of this structural steel fabrication shop, the P-Trak confirmed shop areas with high levels of particulates. It also allowed the determination of processes and/or machinery generating high levels of particles. This is an inexpensive, temporally efficient, and user-friendly method to supplement the qualitative construction of similar exposure groups. This method is not meant to replace a regular monitoring program, where full shift samples are collected and analyzed for various chemicals, but simply to expedite the development of an exposure monitoring program.

## Figures and Tables

**Table 1:** Preliminary Similar Exposure Groups Determined Using Physical Demand Analysis. CNC/PC: computerized numerical control/personal control.

Exposure Classification	Job Titles
Negligible	Shipping Clerk, Yard Man, Building Maintenance Operator, Truck Driver, Mechanics, Mod Yard Crane Operator
Indirect intermittent	Production Manager, Corporate Maintenance Manager, General Foreman, Ironworker
Indirect ambient	Electrician, Tool Crib Attendant, Millwright, Crane Operator
Direct proximal (process)	CNC/PC Operator, Robotic Welding Operator, Shipper, Milling Machine Operator, Wheel Obrator Operator, Descaling Equipment Operator, Lead Hand/Foreman, Fitting Inspector
Direct task	Trade Helper, General Helper, Welding Apprentice, Fitting Apprentice, Ironworker
Direct job	Welder, Fitter, Spray Painter, Sand Blaster, Welding Apprentice, Fitting Apprentice

**Table 2:** Geometric mean and 95% confidence intervals for manual tasks, machine processes, and shop surveys.

		*n*	Geometric Mean (95% CI) (part/mL)
Manual Tasks	All welding activities ^1^	85	150,153 (134,442–167,701)
	Grinding	33	218,992 (189,749–252,741)
	Gouging	20	398,082 (329,304–481,225)
	Oxycutting	38	215,541 (179,945–258,179)
	Drilling ^2^	66	249,584 (207,391–300,360)
	CNC/PC operator ^3^	166	125,317 (112,153–140,026)
Machine Processes	Welding Robot Operator	47	39,274 (35,733–43,167)
	Milling Machine Operator	23	59,328 (55,944–62,917)
Shops	Paint	757	53,999 (51,496–56,624)
	Detail	2732	79,869 (78,179–81,596)
	E	1205	92,906 (89,338–96,618)
	Ironworkers	538	15,441 (14,045–16,976)
	Maintenance	630	37,207 (35,285–39,234)
	Shops A/B/C/D ^4^	5720	64,533 (63,659–65,419)

^1^ Includes data for FCAW, SMAW, and MCAW, ^2^ Data from drilling process from beamlines, ^3^ Includes data from all beamlines, burner table, and CNC plasma, ^4^ Includes data from shops A, B, C, and D.

**Table 3:** Particle Concentration Per Task and Estimated Concentrations Per Job Title.

Job Title	Time Spent on Tasks (%)	Particle Concentration/Task (part/mL)	Estimated Concentration (part/mL)
General Helper	Cutting (10%)	21,554	95,080
	Grinding (10%)	21,899	
	Shop (80%)	51,626	
Trade Helper	Cutting (10%)	21,554	159,492
	Drilling (10%)	24,958	
	Grinding (30%)	65,698	
	Welding (10%)	15,015	
	Shop (50%)	32,267	
Fitter	Drilling (5%)	12,479	133,707
	Grinding (5–35%)	43,798	
	Cutting (5–15%)	21,554	
	Welding (10–15%)	18,769	
	Shop (57.5%)	37,106	
Fitting Apprentice	Same as Fitter	133,707	133,707
Welder	Welding (30–40%)	52,554	109,784
	Grinding (10–15% )	27,374	
	Gouging (5–10%)	29,856	
	Shop (45%)	29,040	
Welding Apprentice	Same as welder	109,784	109,784
Sandblaster/Descaling Equipment Operator	Sandblasting (50%)	NM	NM
	Operating abrasive recycler (20%) Shop (30%)		
CNC/PC Operator	Operating machines (100%)	125,317	125,317
Fitting Inspector	Shop (100%)	64,533	64,533
Crane Operator	Shop (100%)	64,533	64,533
Shipper	Shop (100%)	64,533	64,533
Robotic Welding Operator	Welding robot (100%)	39,274	39,274
Milling Machine Operator	Milling machine (100%)	59,328	59,328
Tool Crib Attendant	Shop (100%)	64,533	64,533
Spray Painter	Paint (100%)	NM	NM
Maintenance	Mechanics–Outside (90%)	NM	NM
	Electricians and Millwrights–Shop (100%)	37,207	37,207
Ironworker	Thermal cutting and welding (5–30%)	33,579	45,932
	Shop (80%)	12,353	
Building Maintenance Operator	Office (80%)	NM	NM
Lead Hand/Foreman	Shop (100%)	64,533	64,533
General Foreman	Offices (90%)	NM	NM
Corporate Maintenance Manager	Office (90%)	NM	NM
Production Manager	Office (90%)	NM	NM
Wheelabrator Operator	Shop (100%)	64,533	64,533

When a range was given for percentage spent on time, the average value was used. NM: not measured.

**Table 4:** Final Similar Exposure Groups.

Exposure Classification	Particle Concentration (part/mL)	Type of Exposure	Job Titles	Old Classification
Very Low to Low Exposure	NM *	Mixed particles	Production Manager	Indirect intermittent
			Corporate Maintenance Manager	Indirect intermittent
			General Foreman	Indirect intermittent
			Building Maintenance Operator	Negligible exposure
			Mechanics	Negligible exposure
			Shipping Clerk	Negligible exposure
			Yard Man	Negligible exposure
			Truck Driver	Negligible exposure
			Mod Yard Crane Operator	Negligible exposure
Medium Exposure	10,000–70,000	Metal particles	Wheel Obrator Operator	Direct proximal
			Lead Hand/Foreman	Direct proximal
			Ironworker	Indirect intermittent/Direct task
			Milling Machine Operator	Direct proximal
			Robotic Welding Operator	Direct proximal
			Burner Table Operator	Direct proximal
		Mixed particles	Electrician and Millwright	Indirect ambient
			Tool Crib Attendant	Indirect ambient
			Shipper	Direct proximal
			Fitting Inspector	Indirect proximal
			Crane Operator	Indirect ambient
High Exposure	>70,000	Metal particles	CNC/PC Operator	Direct proximal
			Welding Apprentice	Direct task/Direct job
			Welder	Direct job
			Fitting Apprentice	Direct task/Direct job
			Fitter	Direct job
			Trade Helper	Direct task
			General Helper	Direct task
Specific Exposure	NM *	Solvents	Spray Painter	Direct job
		Crystalline silica	Sandblaster/Descaling Operator	Direct job/Direct proximal

* NM: not measured.

## Appendix A

**Table A1:** Detailed information about job titles, field observations and interview notes.

Job Title	Observation and Interview Notes
General Helper	General Helper provides miscellaneous help to tradesmen as directed by the foreman. General Helpers are often do detail “running” which is to ensure that assemblies have isometric drawings. They perform housekeeping, crane operation, materials handling and some cutting and grinding. In shop D they do manual “touch-ups” for the paint shop of areas missed by spray painting.
Trade Helper	Trade Helpers aid welders and fitters to perform their tasks more efficiently. Trade helpers perform manual tasks including cutting and grinding in manual detail sections. The role is to perform preparatory work for fitters, so the fitters are less burdened by very basic tasks. They work predominantly in the manual detail areas in each shop, the largest of which is in shop D.
Fitter	The fitters’ main role is to assemble the components prior to welding according to the blueprints. The variability in these exposure generating tasks is due to the type of work in each shop. Fitters weld with SMAW, however doing tack welds is very short lived, only a few seconds. So brief that it was difficult to grab samples with the P-Track, as the delay from pressing the button to sampling could result in missing the 10 s sample. Thus their primary source of particulate generation is thermal cutting with hand-held oxyacetylene torches. The variability in tasks is due to the type of work; more complicated fit-ups will spend more time aligning and clamping than simpler fit-ups. That said, more experienced fitters are generally more productive.
Fitting Apprentice	Job details do not vary significantly from Journeyman Fitter. Like welding apprentices, fitting apprentices fulfill the same role as Journeymen apprentices, it is often that the complexity of the fit-up that varies. 1st year apprentices may be reassigned to other roles, if there is an interim position to be filled.
Welder	The welders’ role is to fuse the metal together according to specification. The variability in these aerosol generating tasks is due to the type of work in each shop. Fluxed Core Arc Welding for a minority of welding, including position welding; Metal Core Arc Welding when possible because of the deposition rate. MCAW can only be welded in Flat position (1G, 1F) therefore it could result in greater exposure in comparison to other positions, respirator policy notwithstanding.
Welding Apprentice	Job details do not vary significantly from Journeyman welder. Most of the welding apprentices perform the same duties as the journeymen welders; it is often the difficulty of task that varies. However, 1st year apprentices have been used in other roles, i.e., crane operator.
Sandblaster/Descaling Equipment Operator	Operates the sandblaster in the sandblast bay, the abrasive recycler and the hopper. This job duties specific to sandblasting do not fill an entire shift; often the sandblaster acts as a general helper in the paint shop and shop D, helping position materials, doing paint touch-ups, cleaning, et cetera.
CNC/PC Operator	Includes the Burner Table; Plasma-Drill (2), Plasma-Punch, Beamline (4), Angle Line, Peddiwriter, Bandsaw. The operators program the computer, moves materials by crane. They also approach the work pieces to mark them with identifiers and heat numbers (15–20%) of the time. The beamline operators clean the cutting area with an air hose, and mark the pieces with a paint pen during operation; they often complain about black smoke–possibly burnt cutting oil-being emitted. Marking the cut pieces brings the operator closer to the process during both the sawing and drilling process. The Plasma table operators often have to approach the machine during operation to mark and removed finished pieces before they fall to the floor, which increases their proximity to the cutting surface.
Fitting inspector	Checks fitting work for specifications and dimensions before welding. Not a defined position on the roster, the two on day shift hold the titles of crane operator and lead hand–quality control (QC) is generally performed by the fitting foremen.
Crane Operator	Movement of materials, positioning of assemblies. They are in charge of the large cranes to move materials the length of the building. Smaller ‘jib‘ cranes move materials locally and are used by tradesmen to reposition material. This position is often filled by those with other job titles, including apprentices, Trade helpers and General helpers, as evidenced by no employed crane operators on weekends.
Shipper	Loads shipments, this involves stacking loads on flatbed trucks and ensuring all items on manifest are present. They also operate fork lifts, in shop D they do “touch-ups” for the paint shop doing manual painting of areas missed by spray painting. Dunnage are wood lengths used to position and support stacked items on trailers.
Robotic Welding Operator	Secures materials, programs the pattern for the robot, observes welding to ensure correct pattern. This system is “offline” meaning that the once the operator is confident in the replicability of the program–he generally observes about 5 runs–he is able to retire to his office to complete paperwork and cue up the program. However, he is able to observe the process in close proximity, and does so, to make minor corrections to the pattern using the remote; herein lies a potential for task exposure.
Milling Machine Operator	Not a defined role, there is a daytime milling machine operator and night shift there is a fitter from shop C to run the milling machine when necessary. Duties include workpiece setup and machine operation.
Tool Crib Attendant	Distributes goods from the tool crib straddling Shop B and Shop C, and repairs hand tool such as grinders. The tool crib is enclosed but is supplied air through the clean air intake on the roof that supplies air to the shops. The tool crib was observed to be a modified work area for a general helper from the paint shop, upon learning of her pregnancy.
Spray Painter	The Spray Painter positions and paints fabricated parts. Wear fitted supplied cartridge style half masks with particulate and VOC filters. They also mix paint and load the paint gun, during these activities they are not required to wear masks.
Maintenance	Includes Millwrights, HD Mechanics, and Electricians, their primary role is to repair machinery in the Shop including Cranes, CNC/PC operated machines. They also repair and upgrade electrical systems. Work primarily in the fabrication shops (>50%).. They also operate manlifts and forklifts, which are sources of particulate as they are diesel powered.. The Mechanics work primarily on vehicles that are largely outside, and prefer not to enter the shop. Both millwrights and electricians work primarily on the shop floor as the machinery is much too large to be moved to the maintenance shop.
Ironworker	Fabricate, Hoist, Install and repair structural ironwork. They also operate man-lifts and forklifts, which are sources of particulate as they are diesel powered. They perform structural maintenance on the shops, for instance they installed the overhead flap for the paint shop and also repair cranes. Their shop does have a welding machine in it but no particle generating activities were observed in the shop.
Building Maintenance Operator	Performs maintenance on the building, including dry-walling, painting, and other handyman type projects. A more descriptive title is handyman. He stores materials in the paint shop office that requires him to cross the shop floor, however is not on the shop floor for significant periods of time, primarily in the shop office and adjoining office building performing his tasks.
Lead Hand/ Foreman	This is a supervisory/QC role. Direct supervision of the shop and verifies production quality. Spends majority of time on the floor, and is largely responsible for QC of their respective shops. Lead Hand, Foreman is an administrative distinction, not an operational one, they perform the same duties.
General Foreman	Most senior floor management; reports to the production manager, day to day operations manager. Tours the floor periodically throughout the day, mostly to observe operations or to check on a specific job order. He is often in the office as his role is more administratively intense.
Corporate Maintenance Manager	Manages the maintenance crew and troubleshoots difficult repairs. Organizes routine maintenance, repairs, and equipment and structural upgrades. Spends about 90% of the time in the office, according to his self-estimate.
Production Manager	In charge of floor operations, reports to VP fabrication. Periodically spends time on the floor, occasional walkthroughs, mostly on floor to address a specific issue that has been raised by the shop foremen.
Wheel Obrator Operator	Loads assemblies to be blasted, adds consumable as needed, removes spent consumable, and runs wheel obrator.
